# Accuracy of plasma Aβ40, Aβ42, and p-tau181 to detect CSF Alzheimer’s pathological changes in cognitively unimpaired subjects using the Lumipulse automated platform

**DOI:** 10.1186/s13195-023-01319-1

**Published:** 2023-10-02

**Authors:** Francisco Martínez-Dubarbie, Armando Guerra-Ruiz, Sara López-García, Carmen Lage, Marta Fernández-Matarrubia, Jon Infante, Ana Pozueta-Cantudo, María García-Martínez, Andrea Corrales-Pardo, María Bravo, Marcos López-Hoyos, Juan Irure-Ventura, Pascual Sánchez-Juan, María Teresa García-Unzueta, Eloy Rodríguez-Rodríguez

**Affiliations:** 1 Neurology Service, Marqués de Valdecilla University Hospital, Avda. de Valdecilla 25, Santander, Cantabria 39008 Spain; 2 Institute for Research Marqués de Valdecilla (IDIVAL), Santander, Cantabria 39011 Spain; 3 Biochemistry and Clinical Analysis Department, Marqués de Valdecilla University Hospital, Santander, Cantabria 39008 Spain; 4 Atlantic Fellow for Equity in Brain Health, Global Brain Health Institute, University of California, San Francisco, San Francisco, USA; 5 CIBERNED, Network Center for Biomedical Research in Neurodegenerative Diseases, National Institute of Health Carlos III, Madrid, 28220 Spain; 6 Medicine and Psychiatry Department, University of Cantabria, Santander, Spain; 7 Universidad Europea del Atlántico, Santander, Spain; 8 Immunology Department, Marqués de Valdecilla University Hospital, Santander, Spain; 9 Molecular Biology Department, University of Cantabria, Santander, Spain; 10 CIEN Foundation/Queen Sofia Foundation Alzheimer Center, Madrid, 28220 Spain

**Keywords:** Alzheimer’s disease, Plasma biomarkers, Early diagnosis, Screening, Validation, Lumipulse

## Abstract

**Background:**

The arrival of new disease-modifying treatments for Alzheimer’s disease (AD) requires the identification of subjects at risk in a simple, inexpensive, and non-invasive way. With tools allowing an adequate screening, it would be possible to optimize the use of these treatments. Plasma markers of AD are very promising, but it is necessary to prove that alterations in their levels are related to alterations in gold standard markers such as cerebrospinal fluid or PET imaging. With this research, we want to evaluate the performance of plasma Aβ40, Aβ42, and p-tau181 to detect the pathological changes in CSF using the automated Lumipulse platform.

**Methods:**

Both plasma and CSF Aβ40, Aβ42, and p-tau181 have been evaluated in a group of 208 cognitively unimpaired subjects with a 30.3% of *ApoE*4 carriers. We have correlated plasma and CSF values of each biomarker. Then, we have also assessed the differences in plasma marker values according to amyloid status (A − / +), AD status (considering AD + subjects to those A + plus Tau +), and ATN group defined by CSF. Finally, ROC curves have been performed, and the area under the curve has been measured using amyloid status and AD status as an outcome and different combinations of plasma markers as predictors.

**Results:**

Aβ42, amyloid ratio, p-tau181, and p-tau181/Aβ42 ratio correlated significantly between plasma and CSF. For these markers, the levels were significantly different in the A + / − , AD + / − , and ATN groups. Amyloid ratio predicts amyloid and AD pathology in CSF with an AUC of 0.89.

**Conclusions:**

Plasma biomarkers of AD using the automated Lumipulse platform show good diagnostic performance in detecting Alzheimer’s pathology in cognitively unimpaired subjects.

**Supplementary Information:**

The online version contains supplementary material available at 10.1186/s13195-023-01319-1.

## Background

The development of novel disease-modifying therapies for Alzheimer’s disease (AD) requires the implementation of biomarkers in clinical practice. To use them on a large scale, it is essential that they can be obtained in a cheap and non-invasive way, similar to screening strategies for other conditions such as cancer or cardiovascular pathology. This is critical, as AD is the leading cause of dementia, and due to the increasing aging of the population, its prevalence is expected to exceed 152 million cases by 2050 [1].

AD is biologically characterized by the cerebral deposition of extracellular amyloid plaques and intraneuronal neurofibrillary tangles formed by phosphorylated tau protein. These pathological alterations occur decades before the onset of the symptoms [2] and are the targets of many of the treatments that are currently under research or in the process of being approved. These changes can be accurately detected through cerebrospinal fluid (CSF) measurements or functional neuroimaging, such as positron emission tomography (PET) [3, 4]. However, these techniques are expensive, invasive, and often unavailable. For these reasons, its scalability to daily practice is currently a challenge. In recent years, technical advances allowing the identification of these markers in the plasma are promising a paradigm shift in AD diagnosis as they open up the possibility of early detection on a large scale [5]. One of the main limitations is that CSF and plasma samples for AD diagnosis are highly susceptible to errors and biases [6, 7], requiring protocolized and standardized pre-analytical handling (collection and storage) so that their results can be generalized and compared between centers.

Alterations in plasma levels of amyloid-beta 1–40 (Aβ40), amyloid-beta 1–42 (Aβ42), or phosphorylated tau protein (p-tau) in different regions such as in threonine 181 (p-tau181), 217 (p-tau217), or 231 (p-tau231) have been demonstrated to be AD-specific and serve as early markers [8–11]. However, even though their detection is now technically possible, they need to be validated to be useful in a clinical setting. To do so, their performance must be tested by comparing them with other known validated biomarkers, such as those in CSF, and this should be done in the clinical and preclinical stages of the disease [12]. The latter will be of great relevance, since it is at that time that disease-modifying drugs, especially anti-amyloid, are expected to be more effective.

Fujirebio’s Lumipulse G immunoassay is a fully automated platform largely validated for CSF biomarker analysis, even in the preclinical stages of AD [13–15]. Moreover, its use for plasma markers is widely available and has shown good results in clinical samples spanning the AD continuum [16]. With all this in mind, the aim of our work is to assess the performance of the Lumipulse assay of p-tau181, Aβ40, and Aβ42 in plasma to detect AD pathological changes on CSF in a cohort of subjects without cognitive impairment (CU).

## Material and methods

### Participants

This research has been carried out with subjects of the “Valdecilla Cohort for the study of memory and brain aging” from the Memory Unit of the Marqués de Valdecilla University Hospital (Santander, Spain). The characteristics of the project have been described in other articles [17], but in brief, it is a prospective cohort designed to longitudinally study the preclinical phases of AD. It is composed of Caucasian CU volunteers. The inclusion criteria are (1) age ≥ 55 years, and (2) signed consent for the extraction and storage of biological samples. The exclusion criteria are (1) cognitive impairment (Clinical Dementia Rating (CDR) [18] > 0), (2) major systemic or psychiatric disease, (3) major sensory deprivation, and (4) contraindications for performing the complementary tests.

In an initial assessment, all participants undergo a lumbar puncture (LP) and blood extraction for measuring Aβ42, Aβ40, p-tau181, and total tau (t-tau) levels. A cranial magnetic resonance imaging, fluorodeoxyglucose PET, and a comprehensive neuropsychological (NPS) study are also performed at baseline. Follow-ups consist of blood extraction and NPS assessment and are performed annually. Two hundred eight subjects were studied for both CSF and plasma Aβ42, Aβ40, and p-tau181 levels (see Additional file 1 for further information).

### Cognitive evaluation

Participants undergo a comprehensive neuropsychological evaluation with sensitive tests that assess all cognitive domains, the details have been described previously [17]. The Mini-Mental State Examination (MMSE) [19] is employed for the global cognitive assessment, and the global CDR score is used to establish the degree of dementia based on functionality and cognition.

### *ApoE* status

We determined the apolipoprotein E (*ApoE*) genotype using TaqMan single nucleotide polymorphism genotyping assay (Applied Biosystems, Foster City, CA, USA). Those subjects carrying ≥ 1 copy of the ε4 allele were considered ε4 + , and the remaining were considered ε4 − .

### Sample pre-analysis

Both CSF and plasma extractions were performed the same day, early in the morning (at 9–10 AM), with a difference of less than 30 min between them. Our hospital is part of the Alzheimer’s Association Quality Control program and complies with the international recommendations for sample collection and storage [20, 21]. The LP was performed with the subject fasting, using a standard 22-G needle, in lateral decubitus, between the L3 and L5 spaces. The CSF was deposited into 15-ml polypropylene tubes and centrifuged at room temperature at 2000* g* for 10 min. The resultant was aliquoted in volumes of 500 µl into 1-ml tubes and then frozen at − 80 °C until analysis in our hospital’s immunology laboratory.

Plasma samples were obtained following the standardized operating procedure described in other works [22]. The blood was stored in 10-ml EDTA tubes and kept cold until processing within the next 3 h. The samples were then centrifuged for 10 min at 1800* g*, and the supernatant was stored in polypropylene tubes in 500 µl volumes and frozen at − 80 °C until analyzed in our hospital’s biochemistry laboratory.

### CSF and plasma biomarkers

CSF Aβ, p-tau, and t-tau values were determined using the automated immunoassay analyzer Lumipulse G600 II [23] (Fujirebio Diagnostics, Malvern, PA, USA), with the kits *Lumipulse G β-Amyloid 1–40* (lot 4YX3085), *Lumipulse G β-Amyloid 1–42* (lot 7ZX3084), *Lumipulse G p-tau181* (lot 5DX3055), and *Lumipulse G t-tau* (lot 6BX3064). We used an unbiased Gaussian mixture modeling based on our sample to establish the cutoff points [24]. For Aβ40, the lower limit of detection (LLD) was 2.78 pg/ml. Intra- and inter-assay variation was < 4.5 and < 7.1%, respectively. Regarding Aβ42, the sensitivity was 150 pg/ml, and intra- and inter-assay variations were < 4 and < 5.9%, respectively. The LLD for t-tau and p-tau181 were 80 pg/ml and 8 pg/ml, respectively. Intra-assay variations were < 1.2 and < 1.5%, and inter-assay variations were < 1.3 and 2.6%, respectively.

Based on this analysis, subjects were categorized according to the ATN classification [25]. We dichotomized these continuous variables and considered Aβ-positive (A +) when CSF Aβ42/40 ratio < 0.076, tau-positive (T +) when CSF p-tau181 > 73.2 pg/ml, and neurodegeneration-positive (N +) if CSF t-tau > 543 pg/ml. Taking these cutoff points, we divided the subjects into those with biologically defined Alzheimer pathology (A + plus T +) as AD + and the rest as AD − .

Plasma Aβ40, Aβ42, and p-tau181 values were also measured using Fujirebio’s Lumipulse G600II with the following kits: *Lumipulse G β-Amyloid 1–40 Plasma* (lot T4B3033), *Lumipulse G β-Amyloid 1–42 Plasma* (lot T6B3074), and *Lumipulse G pTau 181 Plasma* (lot T9B3084). Analytical sensitivities for Aβ40, Aβ42, and p-tau181 were 0.44, 0.37, and 0.052 pg/ml, respectively. Intra-assay variations were < 3.1, < 3.8, and < 2.3%, and inter-run variabilities were < 3.6, < 4.7, and < 3.9%, respectively.

As informed by the manufacturer and following the EP17-A2 CLSI (Clinical and Laboratory Standard Institute) protocol [26], no significant cross-reactivity was found between Aβ40 and amyloid-beta peptides (1–37, 1–38, 1–39, 1–42, 11–40, 17–40, and 1–43) in this immunoassay. Aβ42 assay was also highly specific (no significant cross-reactivity with other amyloid-beta peptides: 1–38 < 0.9% and 1–40 < 1.6%). For p-tau181, minimal cross-reactivity with tau (172–205) amide (0.9%) was found.

### Statistical analysis

We visualized histograms and used the Shapiro test to assess the distribution of the variables. They have been described by mean and standard deviation or median and interquartile range, as appropriate. Plasma and CSF marker levels were log10-transformed for further analysis with parametric tests.

Pearson’s correlation coefficient was used to correlate Aβ40, Aβ42, amyloid Aβ42/40 ratio, p-tau181, and p-tau181/Aβ42 ratio values between plasma and CSF both in the overall sample and stratified by A + and A − groups.

We used ANCOVA to study the potential of plasma markers to detect changes in CSF. Thus, we selected amyloid Aβ42/40 ratio, Aβ40, Aβ42, p-tau181, and p-tau181/Aβ42 ratio as dependent variables; we took amyloid (A + or A −) and tau (T + or T −) as independent variables and age and sex as covariates. We also clustered subjects according to the ATN group and taken into account the A − T − N − , A + T − N − , and A + T + Nx clusters. Since there was only one subject classified as A − T + N − , we excluded it from the group analyses. We assessed whether there were overall differences in these groups using ANOVA, and if differences were found, we performed a post hoc analysis with Tukey’s test to measure the differences between the groups. The effect size of these differences was determined using Cohen’s *d*.

We then performed logistic regression models taking A + / − and AD + / − status as responses and the different plasma markers, age, sex, and *ApoE*4 status, as independent variables. We constructed ROC curves from the results and measured the area under the curve (AUC) to assess the potential of plasma markers as predictors. Optimal cutoff points were estimated using the Youden index, and the DeLong test was used to compare AUCs.

All statistical analyses were performed with the R studio software version 4.2.2.

## Results

### Sample description

We evaluated data from 208 subjects, 136 females (65.4%) and 72 males (34.6%). The median age was 64 years (IQR 60–69). Sixty-three subjects (30.3%) were carriers of at least one *ApoE*4 allele. Biomarker values and distribution according to the ATN group are described in Table 1.

**Table 1:** Sample description

	***n* = 208**
**Characteristics**	
Females, *n* (%)	136 (65.4%)
Age, median (IQR)	64 (60–69)
*ApoE* ε4 carrier, *n* (%)	63 (30.3%)
MMSE (0–30), median (IQR)	29 (28–30)
**CSF biomarkers**	
Aβ40, mean (SD), pg/ml	10,850.7 (3191.4)
Aβ42, median (IQR), pg/ml	819.5 (577–1037)
Ratio Aβ42/40, median (IQR)	0.084 (0.065–0.093)
T-tau, mean (SD), pg/ml	318 (242–400)
P-tau181, median (IQR), pg/ml	37.5 (30.4–54.3)
Ratio p-tau181/Aβ42, median (IQR)	0.042 (0.034–0.061)
**Plasma biomarkers**	
Aβ40, median (IQR), pg/ml	292.8 (266–318)
Aβ42, median (IQR), pg/ml	23.6 (21.5–26.3)
Ratio Aβ42/40, median (IQR)	0.082 (0.074–0.089)
P-tau181, median (IQR), pg/ml	1.1 (0.92–1.36)
Ratio p-tau181/Aβ42, median (IQR)	0.046 (0.038–0.061)
**ATN group, *n* (%)**	
A − T − N −	135 (64.9%)
A + T − N −	50 (24%)
A − T + N −	1 (0.5%)
A + T + Nx	22 (10.6%)

*Abbreviations*: *n* Number of subjects, *IQR* Interquartile range, *MMSE* Mini-Mental State Examination, *CSF* Cerebrospinal fluid, *Aβ* Amyloid beta, *SD* Standard deviation, *T-tau* Total tau, *P-tau* Phosphorylated tau, *A* Amyloid, *T* Tau, *N* Neurodegeneration, *Nx* Both positive and negative neurodegeneration groups

### Plasma and CSF biomarker correlation

**Fig. 1:**
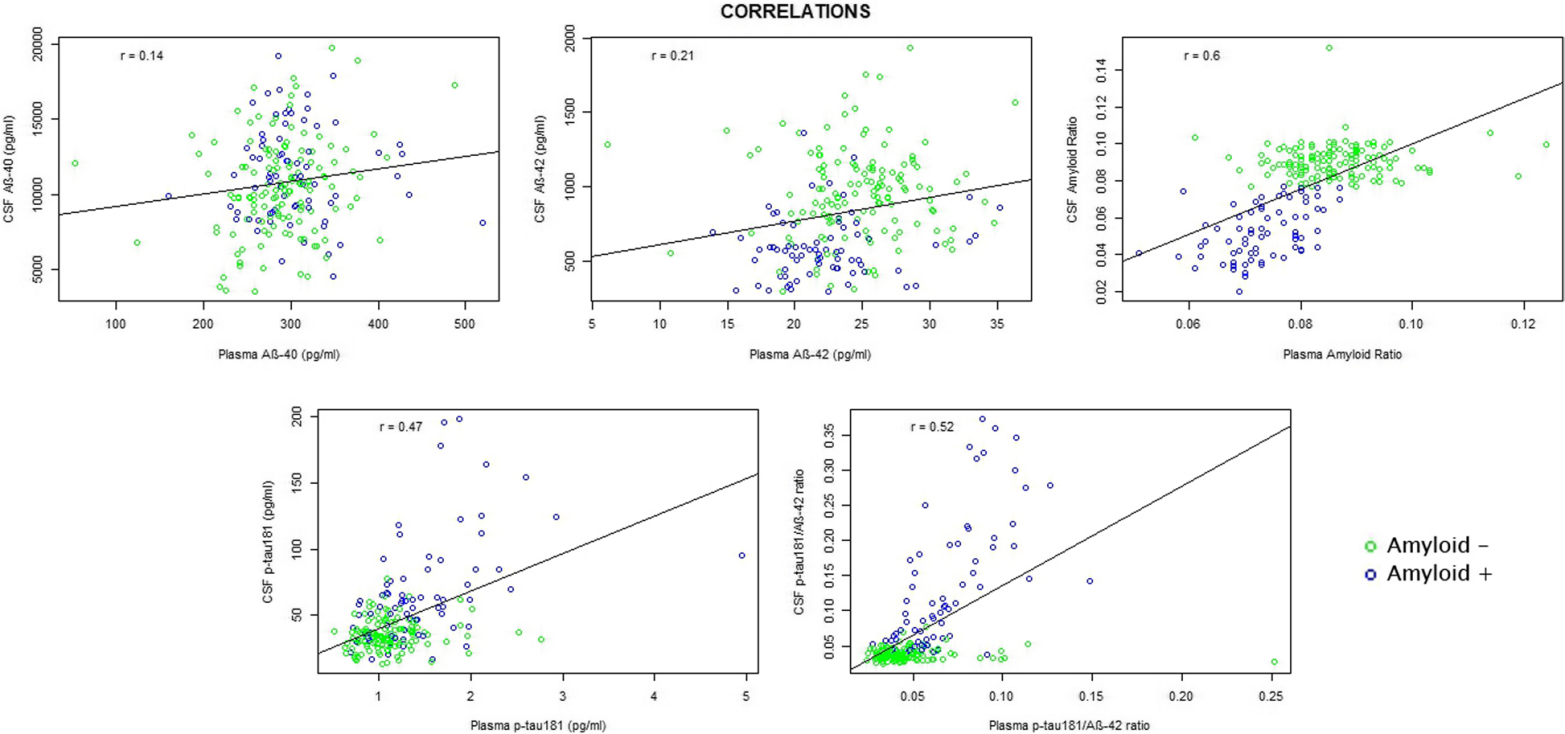
Biomarker correlation between plasma and CSF. The plots show Pearson’s correlation coefficient of biomarkers between plasma and CSF. The ordinate axis corresponds to CSF values and the abscissa axis to plasma values (all, except the amyloid ratio, expressed in pg/ml). The dots represent a pair of values of both variables for each observation. The green ones are those corresponding to amyloid-negative subjects, and the blue ones represent the amyloid-positive subjects. The black line is the regression line. In the upper left corner of each graph is the correlation coefficient (*r*). *Abbreviations*: CSF, cerebrospinal fluid; AB, amyloid β; P-tau, phosphorylated tau

We have correlated the values of different markers between plasma and CSF. With the exception of Aβ40, which showed a marginal correlation (*r* = 0.14; *p*-value = 0.046), the rest of the markers correlated significantly: Aβ42 (*r* = 0.21; *p*-value = 0.002), Aβ42/Aβ40 ratio (*r* = 0.6; *p*-value < 0.0001), p-tau181 (*r* = 0.47; *p*-value < 0.0001), and *p*-tau181/Aβ42 ratio (*r* = 0.52; *p*-value < 0.0001) (Fig. 1).

When stratifying by amyloid status, the correlation between Aβ42 was not significant in either group A − (*r* = 0.06; *p*-value = 0.5) or A + (*r* = 0.14; *p*-value = 0.2). The correlation of amyloid ratio was also not significant in the A − group (*r* = 0.05; *p*-value = 0.54), but it was significant in A + subjects (*r* = 0.45; *p*-value < 0.0001). Something similar happened for p-tau181. Its correlation was not significant in A − subjects (*r* = 0.1; *p*-value = 0.2), but it was significant in the A + group (*r* = 0.44; *p*-value < 0.0001). The correlation of the p-tau181/Aβ42 ratio was also not significant in the A − group (*r* = 0.01; *p*-value = 0.88), but it was in the A + cluster (*r* = 0.66; *p*-value < 0.0001).

### Differences in plasma markers according to amyloid and AD status

**Fig. 2:**
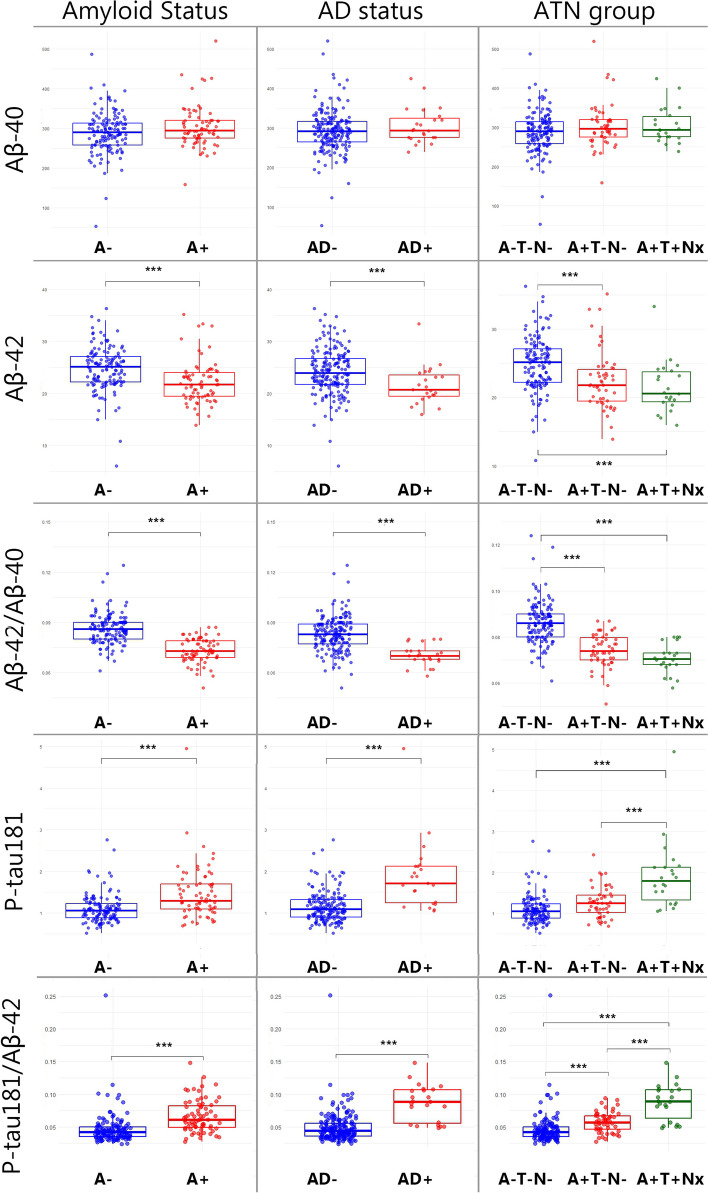
Plasma biomarker values in the A − / + , AD − / + , and ATN groups. The figure shows the box and whiskers plots of plasma markers by groups. The abscissa axis represents the different groups according to the CSF (amyloid status in the first row, AD status in the second one, and ATN group in the third one). The ordinate axis corresponds to plasma concentrations expressed in pg/ml (where applicable). The boxes show the interquartile range (the upper boundary is the Q3, and the lower boundary is the Q1). The line inside the box corresponds to the median of the sample, and the whiskers represent the maximum (upper) and minimum (lower) values. The dots indicate individual values. Significant differences are indicated with a horizontal line and three asterisks between the boxes. *Abbreviations*: AD, Alzheimer’s disease; A, amyloid; Aβ, amyloid beta; P-tau, phosphorylated tau; T, tau; N, neurodegeneration

After that, we studied the mean difference in plasma markers as a function of A and T groups in CSF adjusting for age and sex (Fig. 2). The mean plasma Aβ40 values were not statistically significant between the A + (304.4 pg/ml) and A − (289.1 pg/ml; *p*-value = 0.051) groups nor between AD + (304.8 pg/ml) and AD − (293 pg/ml; *p*-value = 0.26). On the other hand, the mean plasma Aβ42 values were significantly lower in the A + group (22.2 pg/ml) than in the A − group (24.7 pg/ml; *p*-value < 0.0001; Cohen’s *d* = 0.61 with a 95%CI 0.2–1.0), and in the AD + group (21.6 pg/ml) than in the AD − group (24.1 pg/ml; *p*-value = 0.006; Cohen’s *d* = 0.61 with a 95%CI 0.04–1.18).

Similar to plasma Aβ42, the plasma Aβ42/Aβ40 ratio was significantly lower in A + subjects (0.073) than in A − subjects (0.086; *p*-value < 0.0001; Cohen’s *d* = 1.5 with a 95%CI 1.1–2.0). The same happened between the AD + (0.071) and AD − (0.083; *p*-value < 0.0001; Cohen’s *d* = 1.3 with a 95%CI 0.8–1.9) groups.

The mean plasma p-tau181 values were significantly higher in the A + group (1.46 pg/ml) than in the A − group (1.10 pg/ml; *p*-value < 0.0001; Cohen’s *d* = 0.79 with a 95%CI 0.4–1.2). They were also higher in the AD + group (1.87 pg) than in the AD − group (1.15 pg/ml; *p*-value = 0.0004; Cohen’s *d* = 1.7 with a 95%CI 1.1–2.3).

The same happened with plasma p-tau181/Aβ42 ratio values. They were higher in the A + group (0.067) than in the A − group (0.047; *p*-value < 0.0001; Cohen’s *d* = 0.84 with a 95%CI 0.45–1.23) and in the AD − group (0.049) than in the AD + group (0.086; *p*-value < 0.0001; Cohen’s *d* = 1.6 with a 95%CI 1.0–2.2).

### Differences in plasma markers according to ATN classification

We also analyzed the differences between the groups according to the ATN classification (Fig. 2). For plasma Aβ40, we found no differences between the groups (*p*-value = 0.26), so we did not perform further analysis. On the other hand, we found differences between the ATN groups in plasma Aβ42 values (*p*-value = 0.0006). The mean difference between A − T − N − and A + T − N − was 2.26 pg/ml (*p*-value = 0.007; Cohen’s *d* = 0.53 with a 95%CI 0.19–0.86), and between A − T − N − and A + T + Nx subjects, it was 3.23 pg/ml (*p*-value = 0.005; Cohen’s *d* = 0.77 with a 95%CI 0.31–1.23). However, the difference between the A + T − N − and A + T + Nx groups was not significant: 0.96 pg/ml (*p*-value = 0.81).

There were also differences in the overall analysis of Aβ42/Aβ40 ratio between the ATN groups (*p*-value < 0.0001). The mean difference across the A − T − N − and A + T − N − groups was 0.011 (*p*-value < 0.0001; Cohen’s *d* = 1.4 with a 95%CI 1.05–1.76), and across A − T − N − and A + T + Nx, it was 0.016 (*p*-value < 0.0001; Cohen’s *d* = 1.8 with a 95%CI 1.32–2.31). No significant differences were found between the A + T − N − and A + T + Nx groups: 0.003 (*p*-value = 0.31).

Regarding plasma p-tau181 values, we found differences between the ATN groups in the overall analysis (*p*-value < 0.0001), so we analyzed the differences between the groups. The mean difference was 0.16 pg/ml between A − T − N − and A + T − N − subjects, but it was not significant (*p*-value = 0.08). However, it was between the A − T − N − and A + T + Nx groups: 0.81 (*p*-value < 0.0001; Cohen’s *d* = 1.8 with a 95%CI 1.33–2.32), and between A + T − N − and A + T + Nx clusters: 0.64 (*p*-value < 0.0001; Cohen’s *d* = 1.1 with a 95%CI 0.59–1.68).

As for the p-tau181/Aβ42 ratio values, they were also significantly different between the ATN groups (*p*-value < 0.0001). The mean difference between the A − T − N − and A + T − N − groups was 0.01 (*p*-value = 0.02; Cohen’s *d* = 0.49 with a 95%CI 0.15–0.81), between A − T − N − and A + T + Nx 0.04 (*p*-value < 0.0001; Cohen’s *d* = 1.7 with a 95%CI 1.22–2.20), and between A + T − N − and A + T + Nx 0.03 (*p*-value < 0.0001; Cohen’s *d* = 1.5 with a 95%CI 0.95–2.09).

### Performance of plasma biomarkers for preclinical AD diagnosis

Furthermore, we evaluated the ability of different plasma markers to detect Alzheimer’s pathological change (A +) and preclinical Alzheimer’s disease (A + T +) defined by CSF.

**Fig. 3:**
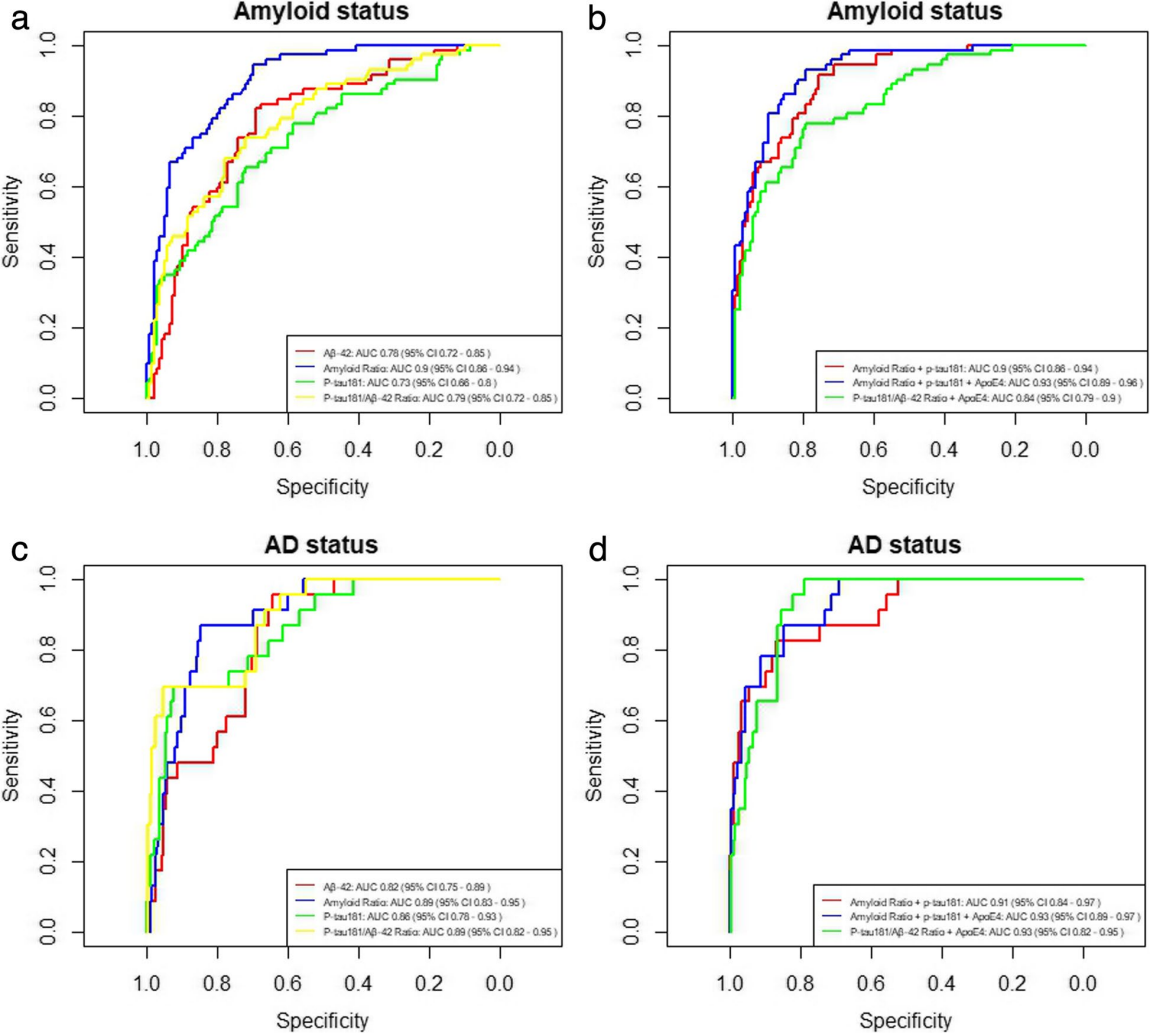
Ability of plasma markers to detect changes in CSF. ROC curves of plasma biomarkers to detect amyloid status both individually (**A**) and combined (**B**) and Alzheimer pathology individually (**C**) and combined (**D**). The abscissa axis shows 1-specificity, and the ordinate axis shows sensitivity. The curves are based on the results of a logistic regression in which different markers and their combinations have been considered (see colors in the legend of each plot), adjusting the results for age and sex. *Abbreviations*: AD, Alzheimer’s disease; AUC, area under the curve; Aβ, amyloid beta; P-tau, phosphorylated tau; CI, confidence interval

When assessing the potential of plasma Aβ42 values to differentiate between A + and A − status, AUC was 0.78 (95%CI 0.72–0.85). The optimal cutoff point of the model was 0.30, with 0.86 sensitivity and 0.52 specificity. Plasma Aβ42/Aβ40 ratio discriminated A + from A − with an AUC of 0.89 (95%CI 0.86–0.94) with an optimal sensitivity of 0.82 and specificity of 0.79 with a threshold of 0.19. P-tau181, on the other hand, showed an AUC of 0.73 (95%CI 0.66–0.80) with optimal sensitivity and specificity of 0.65 and 0.72, respectively (cutoff point 0.34) (Fig. 3A). P-tau181/Aβ42 ratio gave an AUC of 0.79 (95%CI 0.72–0.85) and optimal sensitivity and specificity of 0.68 and 0.78, respectively, with a cutoff point of 0.35.

When AUCs were compared, the amyloid ratio had a significantly higher AUC than that of Aβ42 (*p*-value = 0.0001), p-tau181 (*p*-value < 0.0001), and p-tau181/Aβ42 ratio (*p*-value = 0.0007). However, the Aβ42 AUC was not significantly higher than that of p-tau181 (*p*-value = 0.17) or p-tau181/Aβ42 ratio (*p*-value = 0.87). On the other hand, the p-tau181/Aβ42 ratio showed a greater AUC than p-tau181 alone (*p*-value = 0.0003).

Next, we studied how the combination of different markers predicts amyloid status. When p-tau181 was added to the amyloid ratio in the model, the AUC was 0.9 (95%CI 0.86–0.94), with sensitivity and specificity at the optimal cutoff point (0.27) of 0.92 and 0.75, respectively. When *ApoE*4 status was also added to the model, the AUC increased significantly (*p*-value = 0.02) to 0.92 (95%CI 0.9–0.96), with an optimal sensitivity and specificity of 0.86 and 0.85 at the cutoff point of 0.24, respectively (Fig. 3B). Only the combination of the Aβ42/Aβ40 ratio, p-tau181, and *ApoE*4 status was shown to be significantly better than the Aβ42/Aβ40 ratio alone (*p*-value = 0.02). The combination of the Aβ42/Aβ40 ratio with p-tau181 did not present a significantly higher AUC than the amyloid ratio alone (*p*-value = 0.47).

The combination of p-tau181/Aβ42 ratio and *ApoE*4 status gave an AUC of 0.84 (95%CI 0.79–0.90), a sensitivity of 0.77, and a specificity of 0.79 at the optimal threshold of 0.34. The latter AUC was significantly lower than that of the sum amyloid ratio, p-tau181, and *ApoE*4 (*p*-value = 0.0002) and that of amyloid ratio and p-tau181 together (*p*-value = 0.035).

We also evaluated the ability of plasma markers to differentiate between AD + and AD − subjects both individually (Fig. 3C) and in combination (Fig. 3D). Using Aβ42 plasma individually, the AUC was 0.82 (95%CI 0.75–0.89), with an optimal sensitivity of 0.96 and a specificity of 0.64 at the cutoff point of 0.08. The Aβ42/Aβ40 ratio showed an AUC of 0.89 (95%CI 0.83–0.95) with a sensitivity of 0.87 and a specificity of 0.85 at the optimal threshold of 0.16. P-tau181 had an AUC of 0.86 (95%CI 0.78–0.93) with an optimal sensitivity and specificity of 0.96 and 0.64, respectively, at the 0.2 threshold. P-tau181/Aβ42 ratio gave an AUC of 0.89 (95%CI 0.82–0.95) with sensitivity and specificity of 0.7 and 0.95 at the optimal cutoff point of 0.28.

The AUC of Aβ42/Aβ40 ratio was significantly higher than that of Aβ42 (*p*-value = 0.02), but it was not higher than that of p-tau181 (*p*-value = 0.22) nor that of the p-tau181/Aβ42 ratio (*p*-value = 0.9). P-tau181 and p-tau181/Aβ42 ratio also did not show significantly different AUCs (*p*-value = 0.21), and the same happened between Aβ42 and p-tau181 (*p*-value = 0.33). The AUC of p-tau181/Aβ42 ratio was higher than that of Aβ42 (*p*-value = 0.01).

By adding p-tau181 to the Aβ42/Aβ40 ratio, the AUC increased to 0.9 (95%CI 0.84–0.97) with a sensitivity of 0.83 and a specificity of 0.85 at the optimal cutoff point of 0.14. However, it showed not to be significantly higher than the AUC of amyloid ratio alone (*p*-value = 0.23), but it was higher than that of p-tau181 alone (*p*-value = 0.01). When *ApoE*4 status was added as well, the AUC was 0.93 (95%CI 0.88–0.97), the optimal sensitivity was 0.87, and the specificity was 0.85 at the threshold of 0.12. The AUC was significantly greater than that of the AB42/AB40 ratio alone (*p*-value = 0.004), but it was not significantly higher than that of p-tau181 plus Aβ42/Aβ40 ratio (*p*-value = 0.06). The combination of p-tau181/Aβ42 ratio and *ApoE*4 status gave an AUC 0.93 (95%CI 0.82–0.95) with a sensitivity and specificity of 1 and 0.79, respectively, at the cutoff point of 0.09. However, it was not greater than that of Aβ42/Aβ40 alone (*p*-value = 0.12), that of Aβ42/Aβ40 ratio plus p-tau181 (*p*-value = 0.43), nor that of Aβ42/Aβ40 plus p-tau181 and *ApoE*4 status (*p*-value = 0.8).

## Discussion

With this work, we provide data on the validity of plasma Aβ40, Aβ42, and p-tau181 markers using the fully automated Lumipulse G assay. Our results support the hypothesis that with this platform, widely used in numerous centers worldwide, plasma biomarkers of AD perform consistently well in presymptomatic subjects to detect pathological changes in CSF. Although we lack confirmatory pathological studies, previous research has widely demonstrated the reliability of CSF markers as a reference for AD brain pathology [27–31]. Another point to note is that in our population of cognitively healthy subjects, there is a higher percentage of *ApoE*4 carriers and A + subjects than expected in the general population [32], probably related to volunteer bias.

In this cross-sectional data, we have seen that Aβ42 and p-tau181 values correlate significantly between plasma and CSF samples. When we stratified subjects by amyloid status, some of these correlations disappeared, especially in the A − group, but we suspect that this is due to the small sample size. Moreover, consistent with previous research, in our cognitively asymptomatic cohort, p-tau181 presents higher plasma levels, not only in T + subjects, but also in those A + compared to A − [16, 33, 34]. As expected, the plasma Aβ42/Aβ40 ratio was significantly lower across the ATN spectrum, and p-tau181 levels were progressively higher [35]. As in previous research [36], we have also studied the plasma p-tau181/Aβ42 ratio, and we have found that their levels are significantly different in the amyloid, AD, and ATN groups.

In the possible scenario in which disease-modifying drugs are widely available for presymptomatic phases, the detection of subjects within the AD continuum is essential. Therefore, we have tested the ability of plasma markers to differentiate A + from A − subjects. The individual marker that best predicts amyloid status is the Aβ42/Aβ40 ratio with an AUC close to 0.9. These results place the Aβ42/Aβ40 ratio as a viable screening tool on its own to detect A + subjects, since in the multivariate analysis in which we also took into account p-tau181, the AUC did not improve significantly. For the combination of Aβ42/Aβ40, p-tau181, and *ApoE*4 status, the AUC only improved to 0.93. Considering that this requires measuring two more markers, it does not appear to be a cost-effective model.

The amyloid ratio also provides good results for predicting AD (those subjects with A + and T + markers), with an AUC of 0.89, and does so similarly to p-tau181 which individually presents an AUC of 0.86. As for distinguishing between A − / + subjects, to discriminate Alzheimer’s pathology, the best marker is the Aβ42/Aβ40 ratio alone, as the only model that significantly improves the AUC requires the use of two other markers to increase to 0.93 (the model combining amyloid ratio plus p-tau181 and *ApoE*4 status). This enhancement is not enough to justify its analysis, as it implies performing a further technique, higher costs, and small sensitivity gain. Despite having assessed the cutoff point considered optimal for each model, its use as a screening tool may require the use of other cutoff points that present greater sensitivity, therefore reducing the specificity.

Despite having used different platforms, previous studies have shown results similar to ours, with good performance of the Aβ42/Aβ40 ratio for detecting brain amyloid, with similar AUCs to ours [36, 37]. Other research, however, places the different p-tau species as the most promising single markers for detecting pathology [10, 16, 38]. This difference may be due to the fact that these studies include somewhat different populations, including symptomatic and older subjects than those in our cohort. It is possible that we are analyzing the pathology at an earlier stage of the *continuum* and that can influence the performance of biomarkers.

For the results to be clinically relevant, their robustness is essential, and there are many factors that influence it [39, 40]. We have attempted to minimize biases and errors by using standardized protocols for sample acquisition, processing, and analysis [20–22], but our findings should be replicated in prospective studies, as preliminary studies suggest that findings in fresh and frozen plasma samples are not comparable [6]. The Aβ42/Aβ40 ratio, p-tau181, and p-tau181/Aβ42 ratio effect size have been analyzed, and we have found large effect sizes between the A − / + and AD − / + groups, thus supporting its biological value.

A limitation of our study is the absence of data in other populations such as symptomatic subjects or those with non-Alzheimer’s dementias. Information in this regard is needed to assess the usefulness of this technique to differentiate between pathologies and to monitor the evolution of the disease and its response to treatment. Further efforts should be directed towards longitudinal studies in the community, both in asymptomatic and symptomatic subjects, in order to see the actual impact of plasma markers in clinical practice. Moreover, for screening purposes, positive and negative predictive values should also be evaluated considering the prevalence of AD pathology in the general population and in different age groups.

## Conclusions

In summary, these great AUC values suggest that performing Aβ42, Aβ40, and p-tau18 using Lumipulse assay in CU subjects is a promising screening tool to select subjects from the general population who may require further studies, such as cognitive assessment, PET, and/or CSF in specialized clinics.

### Supplementary Information

**Additional file 1.** Information on the Valdecilla Cohort. Characteristics of the Valdecilla Cohort for the study of memory and brain aging. Recruitment and selection process of the subjects participating in this study.

## Data Availability

The datasets used and/or analyzed during the current study are available from the corresponding author upon reasonable request.

## Abbreviations

A + / −: Amyloid positive/negative
Aβ: Amyloid-β
AD: Alzheimer’s disease
ApoE: Apolipoprotein E
AUC: Area under the curve
CDR: Clinical Dementia Rating
CSF: Cerebrospinal fluid
CU: Cognitively unimpaired
LLD: Lower limit of detection
LP: Lumbar puncture
MMSE: Mini-Mental State Examination
N + / − /x: Neurodegeneration positive/negative/any
NPS: Neuropsychology
P-tau: Phosphorylated tau
PET: Positron emission tomography
Simoa: Single molecule array
T + / −: Tau positive/negative
T-tau: Total tau
