# Supplementary material for: Accuracy of plasma Aβ40, Aβ42, and p-tau181 to detect CSF Alzheimer’s pathological changes in cognitively unimpaired subjects using the Lumipulse automated platform
Source: Alzheimers Res Ther. 2023 Oct 2;15:163. doi: 10.1186/s13195-023-01319-1 (PMC10544460; doi:10.1186/s13195-023-01319-1)
Supplement: Supplementary file 1 — Additional file 1. Information on the Valdecilla Cohort. Characteristics of the Valdecilla Cohort for the study of memory and brain aging. Recruitment and selection process of the subjects participating in this study. [file 13195_2023_1319_MOESM1_ESM.docx]

**Additional File 1**

**Information on the Valdecilla Cohort.**

The ‘Valdecilla Cohort for the study of memory and brain aging’ is set up in the Memory Unit of the Marqués de Valdecilla University Hospital (Santander, Spain). It is a prospective cohort designed to longitudinally study the preclinical phases of AD and its biomarkers. It is an ongoing project whose recruitment began in mid-2018 through an open call in the media of our community. So far, 327 Caucasian subjects have volunteered.

Inclusion criteria are: 1) age ≥55 years; 2) signed consent for the extraction and storage of biological samples. Exclusion criteria are: 1) cognitive impairment (Clinical Dementia Rating (CDR) >0); 2) major systemic or psychiatric disease; 3) major sensory deprivation; 4) contraindications for performing the complementary tests.

All participants undergo an initial assessment with with an extensive questionnaire on demographic characteristics, biometric measurements, family and personal history, as well as a lumbar puncture and blood extraction for measuring Aβ42, Aβ40, p-tau181 and t-tau levels.

In this first evaluation, a comprehensive neuropsychological study is performed by a dementia specialized neuropsychologist. It includes several tests and its scores have been adjusted for age and educational level according to normative data from the NEURONORMA project [1]. Episodic verbal memory is assessed through the Free and cued selective reminding test [2]; the narrative memory is studied through the Logic Memory subtest of the Wechsler Adult Intelligence Scale-IV (WAIS-IV) [3], and the associative memory by using the Spanish validated version of the Face-Name Associative Memory Exam [4]. Rey-Osterrieth Complex Figure Test [5] is used to measure visual recall, and constructive praxis through figure copying.

Categorical (animals) and phonetic (‘p-words’) fluencies were performed to evaluate semantic memory and frontal functions, respectively, and Trail Making Test [6] and Digit Span subtest of the WAIS-IV [3], are also used for assessing frontal functions. Language is measured by using the Boston Naming Test [7].

Processing speed is measured using the Coding subtest of the WAIS-IV [3] and visuoperceptual functions through the Visual Object and Space Perception Battery [8]. We also use the Preclinical Alzheimer's cognitive composite and the Executive Functioning Composite to increase sensitivity in detecting early signs of AD-related cognitive impairment [9,10].

A cranial magnetic resonance imaging (including the Fazekas Score [11] and the medial temporal lobe atrophy measurement [12]) and fluorodeoxyglucose positron emission tomography, are also performed at baseline.

Follow-ups consist of blood extraction and NPS assessment and are performed annually. For this research, two hundred and eight subjects were studied for both CSF and plasma Aβ42, Aβ40 and p-tau181 levels. Details of the selection process can be found in the Additional Figure 1.

**
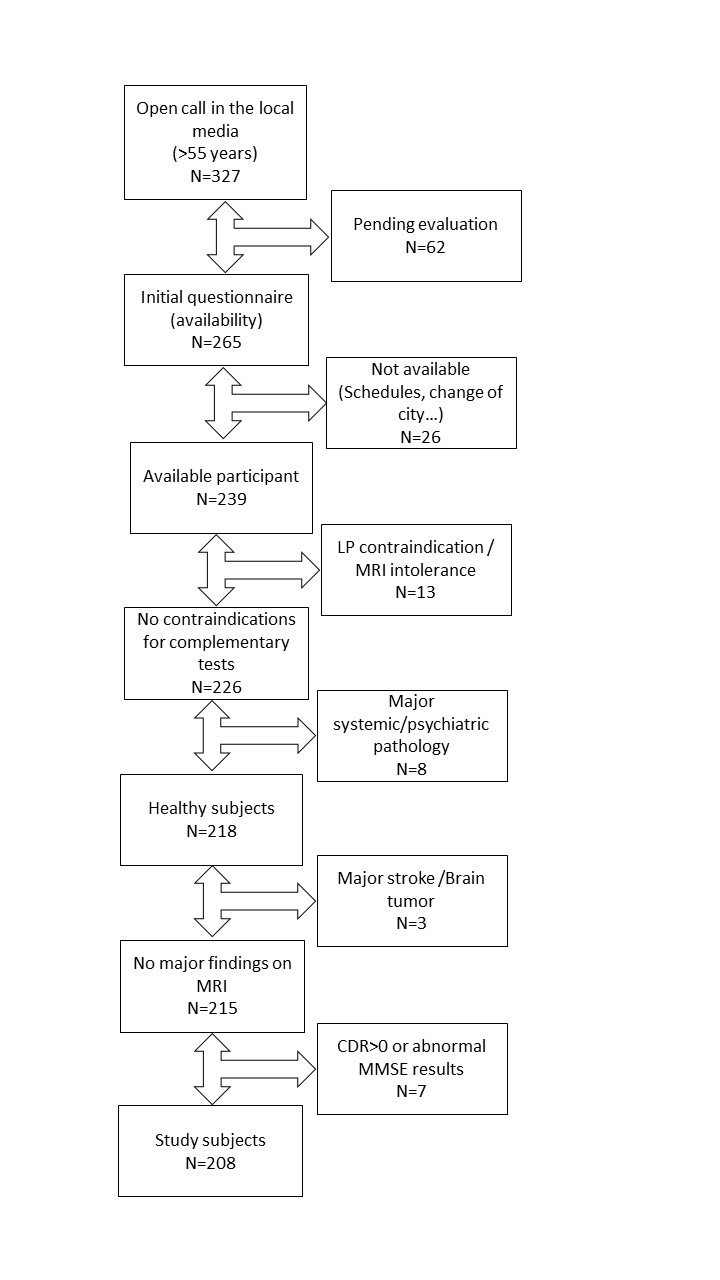
**

Additional Figure 1: Flow chart showing the process of recruitment and exclusion of study subjects. Abbreviations: LP, lumbar puncture. MRI, magnetic resonance imaging. N, number of subjects. CDR, clinical dementia rating. MMSE, mini-mental state examination.

**REFERENCES**

1. Peña-Casanova J, Blesa R, Aguilar M, et al. Spanish Multicenter Normative Studies (NEURONORMA Project): methods and sample characteristics. Arch Clin Neuropsychol. 2009;24(4):307-319. doi:10.1093/arclin/acp027
2. Peña-Casanova J, Gramunt-Fombuena N, Quiñones-Ubeda S, et al. Spanish Multicenter Normative Studies (NEURONORMA Project): norms for the Rey-Osterrieth complex figure (copy and memory), and free and cued selective reminding test. Arch Clin Neuropsychol. 2009;24(4):371-393. doi:10.1093/arclin/acp041
3. Wechsler D. (1945). A standardized memory scale for clinical use. J. Psychol. 19 87–95. 10.1080/00223980.1945.9917223
4. Alegret M, Valero S, Ortega G, et al. Validation of the Spanish Version of the Face Name Associative Memory Exam (S-FNAME) in Cognitively Normal Older Individuals. Arch Clin Neuropsychol. 2015;30(7):712-720. doi:10.1093/arclin/acv050
5. Osterrieth PA (1944). Le test de copie d’une figure complexe [in French]. Archives de Psychologie, 30(30), 206–356.
6. Tombaugh TN. Trail Making Test A and B: normative data stratified by age and education. Arch Clin Neuropsychol. 2004;19(2):203-214. doi:10.1016/S0887-6177(03)00039-8
7. Kaplan E.F., Goodglass H., Weintraub S. The Boston Naming Test. Lea & Febiger; Philadelphia, PA, USA: 1983.
8. Warrington EK, James M (1991) The Visual Object and Space Perception Battery. Bury St Edmunds, England: Thames Valley Test Company.
9. Papp KV, Rentz DM, Orlovsky I, Sperling RA, Mormino EC. Optimizing the preclinical Alzheimer's cognitive composite with semantic processing: The PACC5. Alzheimers Dement (N Y). 2017;3(4):668-677. Published 2017 Nov 10. doi:10.1016/j.trci.2017.10.004
10. Iverson GL, Karr JE, Terry DP, et al. Developing an Executive Functioning Composite Score for Research and Clinical Trials. Arch Clin Neuropsychol. 2020;35(3):312-325. doi:10.1093/arclin/acz070
11. Fazekas F, Chawluk JB, Alavi A, Hurtig HI, Zimmerman RA. MR signal abnormalities at 1.5 T in Alzheimer's dementia and normal aging. AJR Am J Roentgenol. 1987 Aug;149(2):351-6. doi: 10.2214/ajr.149.2.351. PMID: 3496763.
12. Scheltens P, Leys D, Barkhof F, Huglo D, Weinstein HC, Vermersch P, Kuiper M, Steinling M, Wolters EC, Valk J. Atrophy of medial temporal lobes on MRI in "probable" Alzheimer's disease and normal ageing: diagnostic value and neuropsychological correlates. J Neurol Neurosurg Psychiatry. 1992 Oct;55(10):967-72. doi: 10.1136/jnnp.55.10.967. PMID: 1431963; PMCID: PMC1015202.
